# Innovative Chitosan‐Based Formulation for Controlled Release of Enrofloxacin: Pharmacokinetic Analysis in Rabbits

**DOI:** 10.1002/vms3.70618

**Published:** 2025-09-24

**Authors:** Sakineh Khanamani Falahatipour, Ali Rassouli, Hamid Akbari Javar, Yalda Hosseinzadeh Ardakani

**Affiliations:** ^1^ Department of Comparative Biosciences, Faculty of Veterinary Medicine University of Tehran Tehran Iran; ^2^ Department of Pharmaceutics, Faculty of Pharmacy Tehran University of Medical Sciences Tehran Iran

**Keywords:** enrofloxacin, pharmacokinetics, rabbit, sustained release, triple‐layer film

## Abstract

The present study was conducted to evaluate the pharmacokinetics (PK) of a novel triple‐layer formulation of enrofloxacin (ENR) compared to a conventional ENR formulation following subcutaneous (SC) administration in rabbits as an animal model. The triple‐layer formulation comprised chitosan and β‐glycerophosphate (β‐GP) and was cross‐linked with glutaraldehyde. The PK of the conventional ENR formulation was assessed after SC administration at a dosage of 10 mg/kg in rabbits; these results were subsequently compared with the disposition kinetics of the ENR film formulation. High‐performance liquid chromatography (HPLC) was employed to quantify ENR concentrations in plasma, and non‐compartmental analysis was utilized to calculate the PK parameters. The results indicated that the film formulation facilitated sustained drug release. The mean residence time (MRT) for ENR with the film formulation (F1) was significantly enhanced, presenting a 29‐fold increase compared to the conventional formulation (*p* < 0.05). Although the *C*_max_ of the conventional ENR formulation was significantly higher than that of film F1 (by a factor of 18.5), the *T*_max_ value for film F1 was significantly greater than that of the conventional drug, showing an increase of 5.1 times. In conclusion, the triple‐layer film demonstrated favourable characteristics for the sustained delivery of ENR, particularly exhibiting a high MRT. Consequently, the use of films as a delivery system may provide an effective strategy to extend the pharmacological activity of ENR in animals.

## Graphical abstract

This study develops a novel triple‐layer chitosan film for sustained enrofloxacin delivery in rabbits. The implant significantly extends drug residence time (29‐fold) and maintains therapeutic levels for 144 h, offering a promising strategy to reduce dosing frequency in veterinary practice.

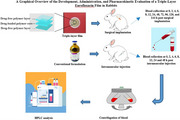

## 1 Introduction

Management challenges in veterinary pharmacology are heightened at two distinct levels. First, pet owners routinely face difficulties in administering oral medications to both feline and canine patients. Second, the restraint of laboratory animals for subcutaneous (SC) or intraperitoneal (IP) injections presents a considerable challenge (Guarnieri et al. 2014). Following conventional administration, the systemic availability of antimicrobial drugs is typically rapid, with a short residence time dictated by the drug's half‐life. This pharmacokinetic (PK) profile necessitates frequent drug dosing during antimicrobial therapy (Kapoor and Katare 2012). Consequently, there is a compelling rationale to explore the potential of sustained‐release drug formulations to optimize clinical efficacy in animals. The use of long‐acting formulations can significantly reduce the frequency with which animals need to be restrained for medication administration (Guarnieri et al. 2014).

Implantable drug delivery systems offer the potential to optimize a medication's therapeutic characteristics, thereby facilitating safe, effective and reliable treatments (Dash and Cudworth 1998). Furthermore, in the event of adverse effects, such systems can be readily removed, providing a reversibility not attainable with depot injections (Metzger et al. 2007). Biodegradable systems, upon depletion of the incorporated drug, degrade into non‐toxic byproducts within the body, thus eliminating the need for surgical removal—in contrast to non‐biodegradable controlled‐release devices (Kapoor and Katare 2012). However, these systems are not without limitations, including potential toxicity, pain associated with implantation and the requirement for microsurgical techniques during implantation (Rajgor et al. 2011).

When a drug is incorporated into a polymer solution, it becomes entrapped within the resulting polymer matrix. Over time, as the polymer undergoes biodegradation, the drug is released (Pandya et al. 2014). Indeed, by employing such systems, the entrapped drugs are formulated to exhibit a controlled and prolonged release profile, thereby extending their duration of action (Rathbone 2012). For controlled SC delivery, the use of biodegradable smart polymer‐based implantable controlled‐release drug delivery systems presents a potentially advantageous approach, addressing the acceptability concerns associated with non‐biodegradable solid implants (Kapoor and Katare 2012). Local antibiotic delivery offers the advantage of achieving high tissue concentrations while maintaining relatively low systemic drug levels, thus mitigating some of the systemic toxicities associated with traditional antibiotic administration (Gitelis and Brebach 2002).

Chitosan, a naturally derived polysaccharide, exhibits considerable promise in pharmaceutical applications owing to its biocompatibility, biodegradability, chemical inertness, low cost, non‐toxicity, hydrophilicity, mechanical strength, solubilization capacity and film‐forming properties (Huang et al. 2002; Kim et al. 2005; Li et al. 2014). The film‐forming characteristics of chitosan, coupled with its mechanical strength and relatively slow biodegradation rate, render it suitable for various drug delivery applications. Drug‐loaded chitosan films represent a novel class of drug delivery systems with potential for sustained local delivery of diverse therapeutic agents (Dhanikula and Panchagnula 2004).

Enrofloxacin (ENR), a fluoroquinolone antimicrobial agent, is exclusively utilized in veterinary medicine. It demonstrates potent bactericidal activity against a broad spectrum of clinically significant Gram‐positive and Gram‐negative pathogens, including *Chlamydiae* and *Mycoplasma*. ENR and its active metabolite, ciprofloxacin, exhibit concentration‐dependent bactericidal effects. Its favourable safety profile, broad‐spectrum activity, low minimum inhibitory concentrations (MICs) and prolonged post‐antibiotic effect contribute to its widespread use in veterinary practice (Scheer 1987).

ENR PK are characterized by high bioavailability across numerous species and rapid absorption following SC, intramuscular (IM) or oral administration. The drug displays excellent tissue penetration, widespread distribution and a serum half‐life ranging from 3 to 6 h (López‐Cadenas et al. 2013; Anadon et al. 1999). All currently available ENR formulations are immediate‐release products, requiring twice‐daily administration for several days or weeks. To reduce dosing frequency, considerable effort has been devoted to developing alternative, sustained‐release ENR formulations (Kumar et al. 2015).

Although chitosan‐based drug delivery systems have been extensively studied, our novel triple‐layer design introduces a breakthrough architecture in which a drug‐loaded core is strategically sandwiched between two rate‐controlling chitosan layers. This innovative configuration fundamentally differs from conventional single‐layer systems by providing dual control mechanisms. Although using additional barrier layers is an efficient method to manipulate drug release profiles and reduce burst release, it is inherently expensive due to the additional materials required and the difficulties associated with controlling barrier quality (Kafshgari Morteza et al. 2011). Our design directly addresses this cost‐quality challenge by employing a single, biocompatible material (chitosan) in a multilayer structure, which simplifies manufacturing and ensures consistent barrier quality while effectively overcoming the key limitations of uncontrolled burst release and insufficient duration of therapeutic drug levels.

This study bridges the critical gap between our prior in vitro demonstration of triple‐layer chitosan film efficacy (Rassouli et al. 2018) and clinical application by conducting the first comprehensive in vivo evaluation. We compared the PK profiles of our proprietary implantable triple‐layer ENR film and a conventional ENR formulation in rabbits, validating its ability to maintain therapeutic drug levels under physiological conditions.

## 2 Materials and Methods

### 2.1 Materials

Medium molecular weight chitosan with a 75%–85% degree of deacetylation (DDA) and β‐glycerophosphate (β‐GP) disodium salt pentahydrate were procured from Sigma‐Aldrich (St. Louis, MO, USA). Acetic acid, glutaraldehyde and glycerol were obtained from Merck (Darmstadt, Germany). Enrovet (ENR aqueous solution, 100 mg/mL) was provided by Aburaihan Pharmaceutical Company (Tehran, Iran), and the ENR standard (99.57% purity) was supplied by TEMAD Pharmaceutical Company (Tehran, Iran). All other chemicals were of reagent grade.

#### 2.1.1 Preparation of the Triple‐Layer Film

Triple‐layer films were fabricated by sequentially casting individual layers in a mould. The first layer was prepared by dissolving 150 mg of powdered chitosan in 4 mL of a 1% (v/v) aqueous acetic acid solution, followed by overnight incubation at 4°C. Subsequently, a solution containing 500 mg β‐GP and 50 µL glutaraldehyde in deionized water was prepared. Both the chitosan and β‐GP solutions were cooled in an ice‐water bath for 15 min before combining. The β‐GP solution was added dropwise to the chitosan solution, followed by the addition of 100 µL glycerol. The resulting 5 mL solution was cast to form the first layer. The third layer was prepared using the same method as the first layer.

The second (middle) layer was prepared by dissolving 100 mg of chitosan in 4 mL of a 1% (v/v) aqueous acetic acid solution. This solution was then mixed with 100 mg of ENR and stirred for 2 h at room temperature. The drug‐loaded second layer was cast onto the centre of the pre‐formed first layer. The first and third layers were allowed to dry for 48 h, whereas the second layer was dried for 24 h (Rassouli et al. 2018). The resulting chitosan‐based films were sterilized by gamma‐ray irradiation (10 kGy) prior to use in rabbits.

### 2.2 PK Study

#### 2.2.1 Animals

Eighteen healthy white albino rabbits weighing 2.8–3 kg were used in this study. The animals were housed under a controlled 12‐h light/dark cycle with free access to water and standard feed. Environmental humidity and temperature were maintained at 45%–65% and 25°C ± 2°C, respectively. The rabbits were monitored for 1 week to assess any clinical signs and ensure acclimatization prior to drug administration.

#### 2.2.2 Drug Administration

The rabbits were randomly assigned to three groups: two test groups (conventional ENR product, *n* = 6; enrofloxacin implant, *n* = 6) and one control group (*n* = 6). The animals had continuous access to water and feed throughout the study. A single SC dose of 10 mg/kg of the conventional ENR product (Enrovet) was administered to the first test group. The second group received a single SC dose of the film formulation equivalent to 33.3 mg/kg of ENR. The implants were sectioned into four pieces prior to administration.

**FIGURE 1:**
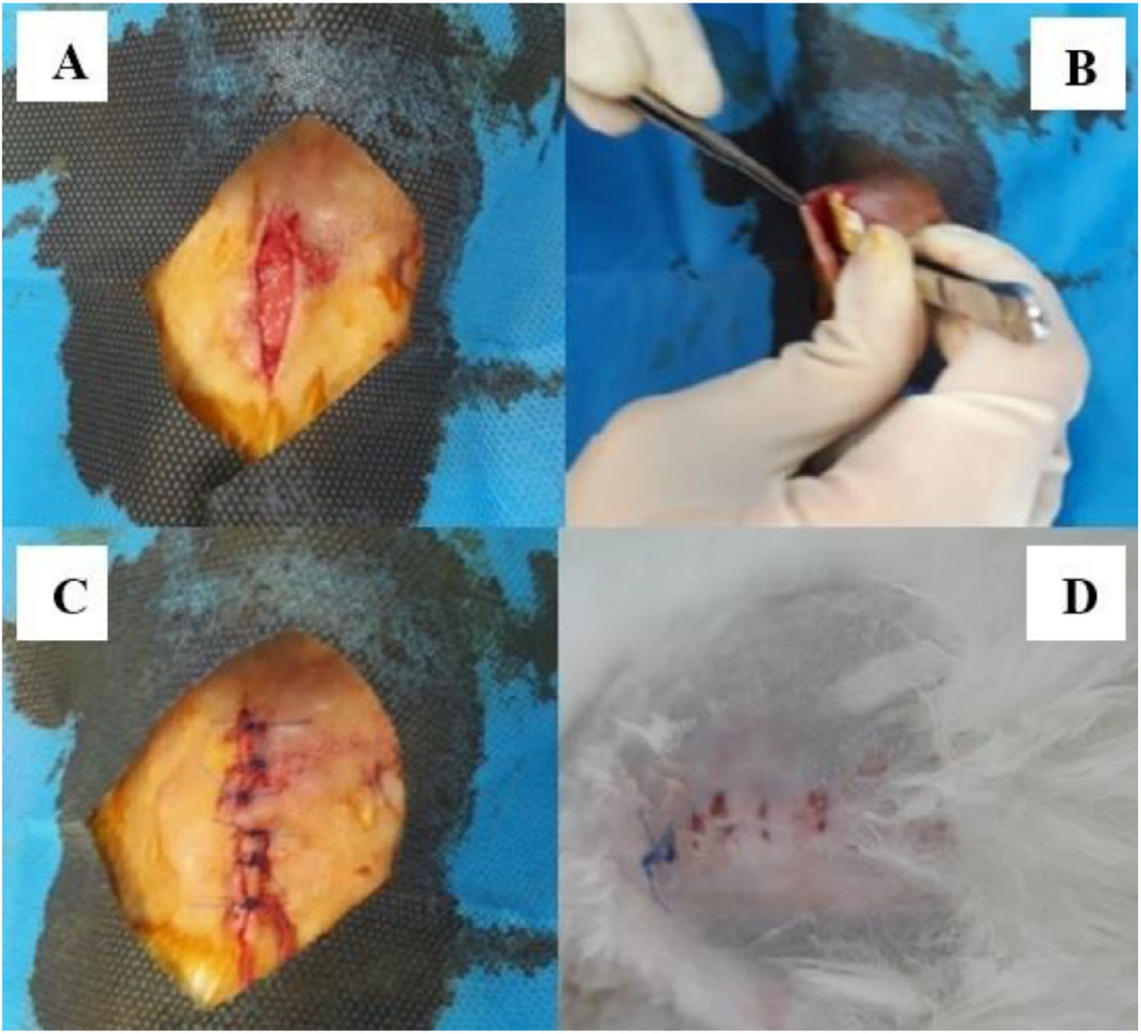
Surgical implantation and wound healing process of the triple‐layer chitosan film. (A) Step 1: Creation of subcutaneous pocket for implantation. (B) Step 2: Precise placement of the sterile film implant. (C) Step 3: Wound closure with interrupted sutures. (D) Postoperative Day 7 showing complete wound healing with no signs of inflammation or implant. The sequential images demonstrate the surgical feasibility and tissue compatibility of the implant system.

All rabbits in the second and control groups were fasted overnight and subsequently anaesthetized via IM injection of xylazine and ketamine for 4–5 h. A small incision was made in the loose skin at the back of the rabbits’ necks for SC implantation following blood sampling at the zero‐time point. The incision was then routinely sutured (Figure 1A–C). The third (control) group received an equal volume of the drug‐free implant formulation (blank).

By postoperative Day 7, complete wound healing was observed with no signs of inflammation, infection or implant extrusion (Figure 1D).

#### 2.2.3 Blood Sampling

Blood samples (approximately 1.5 mL) were collected from the jugular vein of the rabbits into sterile heparinized tubes at predetermined time points. For the first test group (conventional formulation), samples were obtained at baseline (0 h) and at 0.5, 1, 2, 4, 6, 8, 12, 24 and 48 h post‐administration. For the second group (film formulation) and the control group, samples were collected at 0, 2, 4, 6, 8, 12, 24, 48, 72, 96, 120 and 144 h. The samples were centrifuged at approximately 4825 × *g* for 10 min within 1 h of collection, and the resulting plasma was stored at −20°C until analysis.

### 2.3 Analytical Method

For plasma sample preparation, 50 µL of 1 N NaOH was added to 500 µL of each rabbit plasma sample and vortexed for 30 s. Subsequently, 100 µL of perchloric acid and 100 µL of deionized water were added to each sample, followed by vortexing for 1 min and centrifugation at approximately 4825 × *g* for 5 min (Eppendorf Centrifuge, Model 5810 R, Germany). The supernatant was transferred to specific glass tubes, and 20 µL of each sample was injected into the high‐performance liquid chromatography (HPLC) system for analysis.

ENR concentrations in plasma were determined using an HPLC system (Waters, USA) equipped with a UV detector, solvent degasser, multi‐solvent pump, autosampler, interface and Chromate software. A Chromolith‐RP18e column (50 mm × 4.6 mm; Merck, Darmstadt, Germany) was used for separation. The analytical method referenced the technique described by McKellar et al. The mobile phase consisted of 14% acetonitrile, 85% water, 0.4% triethylamine and 0.6% phosphoric acid (35%), filtered through a 0.45 µm membrane filter under vacuum prior to use (McKellar et al. 1999). Chromatographic separation was performed with UV detection at 294 nm and a flow rate of 1 mL/min.

An ENR stock solution (1.0 mg/mL) was prepared by dissolving 10 mg of ENR standard in 10 mL of a 1:1 (v/v) acetonitrile/water mixture. This solution was serially diluted in drug‐free rabbit plasma to obtain final concentrations of 0.1, 0.25, 0.5, 0.75, 1.0, 1.5, 2.0 and 2.5 µg/mL.

The HPLC method for quantifying ENR in rabbit plasma was validated for linearity, precision, accuracy, selectivity, recovery and sensitivity. A standard calibration curve was constructed using eight ENR concentrations (0.1–2.5 µg/mL) and used to determine ENR levels in the plasma samples.

### 2.4 PK Data Analysis

To characterize the ENR plasma concentration–time profile, individual data from each rabbit were analysed. The PK parameters, including the maximum plasma concentration (*C*_max_) and the time to reach *C*_max_ (*T*_max_), were determined directly from the observed concentration–time data. ENR PK analysis was performed using a noncompartmental approach to estimate key parameters, including the area under the concentration–time curve (AUC), area under the first moment curve (AUMC) and mean residence time (MRT). The area under the curve from 0 to 144 h (AUC_0–144_) was calculated applying the linear trapezoidal rule.

The following equation was used to calculate the relative bioavailability (*F*_rel_):

(1) $$F_{\mathrm{rel}}=\frac{\mathrm{AUC}\;\left(0-144\right)\;\mathrm{of}\;\mathrm{film}\;\mathrm{ENR}\;\mathrm{formulation}\times \mathrm{Dose}\;\mathrm{of}\;\mathrm{conventional}\;\mathrm{drug}\;}{\mathrm{AUC}\;\left(0-144\right)\;\mathrm{of}\;\mathrm{conventional}\;\mathrm{drug}\times \mathrm{Dose}\;\mathrm{of}\;\mathrm{film}\;\mathrm{ENR}\;\mathrm{formulation}}$$

### 2.5 Statistical Analysis

Data are presented as mean ± standard deviation (SD) and were analysed using SPSS software (version 19). A one‐way analysis of variance (ANOVA) was used to statistically evaluate the PK parameters, with a significance level set at *p* < 0.05.

## 3 Results

**FIGURE 2:**
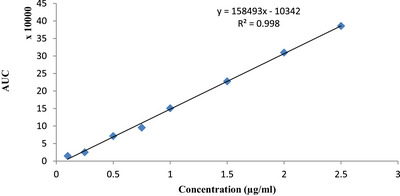
Standard calibration curve of enrofloxacin for HPLC analysis.

For the HPLC analysis of ENR, the calibration curve demonstrated linearity over the concentration range of 0.1–2.5 µg/mL, with a correlation coefficient (*R* ^2^) of 0.998 (Figure 2). The limit of detection (LOD) in rabbit plasma was 0.05 µg/mL, whereas the limit of quantification (LOQ) was established at 0.1 µg/mL. No peak corresponding to the retention time of ENR was observed in chromatograms from control group plasma samples.

**FIGURE 3:**
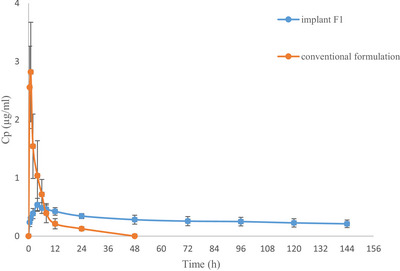
Semi‐logarithmic plot of plasma concentration (Cp) versus time profiles of enrofloxacin after SC use of a single dose of ENR conventional formulation (10 mg/kg) and ENR implant (33.3 mg/kg) in rabbits. Data are expressed as mean ± SD (*n* = 8).

The rabbits exhibited good tolerance to ENR without significant adverse events. The mean plasma concentration–time profiles of ENR following SC administration of the film and conventional formulations in each group are presented in Figure 3. The PK parameters of both ENR formulations are summarized in Table 1.

**TABLE 1:** Pharmacokinetic parameters of enrofloxacin (ENR) in rabbits after subcutaneous (SC) administration of conventional formulation (10 mg/kg) and SC implantation of a single dose of F1 film (33.3 mg/kg body weight).

PK parameters	Conventional formulation	Triple‐layer film	*p* value
**AUC_0–144_ (µg h/mL)**	14.23 ± 3.81	40.94 ± 8.82	<0.01^*^
**AUC_0–∞_**	15.43 ± 4.04	83.19 ± 24.98	<0.01^*^
**AUMC_0–144_ (µg h^2^/mL)**	100.96 ± 61.24	8122.13 ± 2214.88	<0.01^*^
**MRT (h)**	6.77 ± 2.45	196.34 ± 12.82	<0.01^*^
***C*_max_ (µg/mL)**	3.14 ± 0.55	0.55 ± 0.11	<0.01^*^
***T*_max_ (h)**	0.92 ± 0.20	4.67 ± 1.03	<0.01^*^
***F*_rel_ (%)**	100	86.40 ± 18.62	<0.05^*^

*Note*: Data are expressed as mean ± SD (*n* = 6). *C*_max,_ maximal concentration; *F*_rel_, relative bioavailability; *T*_max,_ time to reach *C*_max_.

Abbreviations: AUC, area under the plasma concentration‐time curve; AUMC, area under the first moment curve; MRT, mean residence time.

* Data with significant difference (*p *< 0.05).

The mean maximum plasma concentration (*C*_max_) for the conventional formulation was significantly higher than that of the film formulation (*p* < 0.05). Conversely, the film formulation showed significantly greater values for the time to reach *C*_max_ (*T*_max_), area under the concentration–time curve from 0 to infinity (AUC_0–∞_), MRT and relative bioavailability (Rel. BA) compared to the conventional formulation (*p* < 0.05).

Table 2 displays dose‐normalized PK parameters (AUC_0–144_/dose, AUC_0–∞_/dose, AUMC_0–144_/dose and *C*_max_/dose) to account for dose‐dependent effects (Hsu et al. 1997). This table exclusively presents dose‐normalized parameters, as non‐dose‐dependent metrics (MRT, *T*_max_) and *F*_rel_ (calculated using Equation 1 with inherent dose normalization) require separate consideration in PK evaluation.

**TABLE 2:** Dose‐normalized pharmacokinetic parameters of conventional and triple‐layer film formulations (mean ± standard deviation [SD]).

Parameter	Conventional (10 mg/kg)	Triple‐layer film (33.3 mg/kg)	*p* value
**AUC_0–144_/Dose (h/mL/kg)**	1.423 ± 0.381	1.230 ± 0.265	<0.01^*^
**AUC_0–∞_/Dose (h/mL/kg)**	1.543 ± 0.404	2.498 ± 0.750	<0.01^*^
**AUMC_0–144_/Dose (h^2^/mL/kg)**	10.096 ± 6.124	243.907 ± 66.513	<0.01^*^
***C*_max_/Dose (mL^−1^ kg^−1^)**	0.314 ± 0.055	0.017 ± 0.003	<0.01^*^

*Note*: Data are expressed as mean ± SD (*n* = 6).

Abbreviations: AUC, area under the plasma concentration‐time curve; AUMC, area under the first moment curve; MRT, mean residence time.

* Data with significant difference (*p* < 0.05).

The persistent significant differences (*p* < 0.05) following normalization suggest that intrinsic formulation characteristics, particularly the sustained‐release properties of the triple‐layer film, substantially influence the PK profile independent of the administered dose.

## 4 Discussion

This study successfully bridges the critical gap between in vitro development and clinical application by demonstrating the controlled release of ENR from the triple‐layer chitosan film under physiological conditions. The PK profile confirms the system's stability against biological challenges while maintaining therapeutic drug levels, overcoming a key limitation of conventional sustained‐release systems. These findings advance polymer‐based drug delivery technology by validating chitosan's efficacy as a biodegradable carrier for SC ENR implantation, where regulated drug delivery to target tissues and sustained therapeutic effects are paramount (Kleppner et al. 2006; Lin et al. 2001; Mandal 1999). The successful in vitro‐to‐in vivo translation (Rassouli et al. 2018) establishes this platform's potential for veterinary applications requiring long‐term antibiotic therapy.

Although our previous in vitro studies demonstrated the superior sustained‐release performance of the triple‐layer design compared to single‐layer films (Rassouli et al. 2018), the current in vivo investigation focused exclusively on the optimized three‐layer system to validate its clinical potential. This approach was based on the formulation's demonstrated in vitro advantages, including significantly prolonged release kinetics (168 vs. 5 h) and reduced burst effect compared to conventional single‐layer films (Rassouli et al. 2018). The PK results confirm that our multilayer architecture successfully translates its in vitro performance to biological systems, maintaining stable drug levels without the rapid initial release characteristic of simpler chitosan formulations.

Although evaluated via SC administration in this study, our triple‐layer chitosan film was specifically designed for surgical implantation to prevent postoperative infections. This approach offers critical advantages over systemic antibiotic therapy by creating localized, high‐concentration antimicrobial protection precisely at the wound site—a feature particularly crucial in deep tissue procedures where vascular compromise may limit systemic drug delivery. The film's controlled‐release mechanism maintains therapeutic levels throughout the vulnerable healing period, whereas its complete biodegradability eliminates the need for secondary removal procedures. Unlike conventional sustained‐release systems designed for general administration, our implant integrates seamlessly into surgical workflows, providing targeted prophylaxis exactly where needed. Although further comparative studies with other long‐acting formulations would be valuable, this technology uniquely addresses the specific challenges of surgical site infection (SSI) prevention through its combination of localized delivery and hassle‐free resorption.

In veterinary medicine, it is imperative that formulations of long‐acting injectables and implants incorporate biocompatible and safe excipients (Rathbone 2012). Chitosan‐based formulations exhibit such properties. As a drug carrier, chitosan is characterized by its metabolic degradability within the body, facilitating drug elimination through renal clearance following administration (Saikia et al. 2015). The polymer matrix degrades over time in the aqueous environment surrounding the implant, enabling sustained drug release (O'Neil 2010).

In the current study, the PK of the ENR film were compared with those of the conventional formulation. The results indicated that the novel film formulation prolonged ENR release over a 7‐day therapeutic period, exhibiting sustained‐release characteristics and demonstrating good tolerability when administered alongside local anaesthetic prior to SC insertion. All rabbits exhibited resilience throughout the study, with no clinical signs of toxicity or adverse reactions—such as redness, swelling, infection or implant expulsion—corroborating the system's biocompatibility. Plasma ENR concentrations remained stable yet lower than conventional administration, significantly mitigating the risk of drug toxicity or adverse effects. A key limitation of this study is the absence of histopathological analysis of the implant site, which precludes detailed assessment of local tissue response at the microscopic level. Although macroscopic evaluation revealed no signs of inflammation, infection or implant rejection, future studies should include longitudinal histopathology to evaluate chronic inflammatory responses and tissue‐biomaterial interactions more comprehensively. Additionally, future studies are warranted to determine whether ENR concentrations in the blood of treated animals can achieve therapeutic levels against target pathogens. Such data would provide deeper insights into the long‐term biocompatibility, safety profile and clinical efficacy of the implant system.

The PK advantages of the film formulation arise primarily from its capacity to create a drug depot that facilitates gradual release, thereby maintaining elevated local drug concentrations in surrounding tissues over an extended period (Hoare and Kohane 2008). Typically, the PK profile of a drug is influenced more by the physicochemical properties of the long‐acting formulation than by the inherent properties of the drug itself. These sustained‐release formulations provide numerous benefits (Rathbone 2012), including enhanced PK and pharmacodynamic profiles, an increased therapeutic index, reduced adverse effects and ultimately improved systemic performance (Saikia et al. 2015).

Faridi et al. (2023) synthesized chitosan nanoparticles loaded with ciprofloxacin, reporting 80% drug release within 24 h. In contrast, plasma ENR concentrations from the conventional formulation declined rapidly and became undetectable after 48 h. Conversely, our triple‐layer film formulation demonstrated sustained ENR release for up to 144 h, significantly extending the MRT compared to the conventional formulation, which exhibited an MRT of approximately 6.77 h. The plasma ENR levels following film administration showed a slow elimination pattern lasting over 7 days, attributable to the gradual dissolution of ENR from the film matrix. Another study developed stretchable doxorubicin‐loaded chitosan films using acetic acid, achieving 27% elongation and 70% drug release, demonstrating potential as a drug sealant (Lee et al. 2023). However, our triple‐layer film formulation provides a substantially prolonged drug release profile (up to 144 h) compared to both conventional methods and the 24‐h release observed in nanoparticle systems.

Guarnieri et al. (2014) developed cholesterol‐buprenorphine drug pellets and conducted PK studies in female BALB/c mice, revealing peak buprenorphine levels approximately 8 h post‐implantation, with plasma concentrations becoming undetectable within four to 5 days. Yin et al. evaluated the tissue distribution and PK of sustained‐release cisplatin nanoparticle implants in dogs, reporting rapid systemic distribution with peak platinum levels achieved within 10 min, followed by swift elimination. In comparison, cisplatin nanoparticle implants exhibited prolonged blood circulation and favourable PK properties, attributed to the incorporation of polylactic acid (PLA) (Yin et al. 2015). Similarly, Metzger et al. (2007) developed implantable haloperidol formulations using biodegradable polymers and assessed the long‐term in vivo PK profile of PLA implants in rabbits, noting detectable serum levels for 360 days with a mean concentration of 6.6 ± 1.1 nmol/L (2.5 ± 0.4 ng/mL). A study highlighted chitosan's efficacy as a localized antibiotic delivery system, demonstrating sustained release of amikacin (96.23% at 72 h) and daptomycin (88.55% at 72 h). The eluted antibiotics effectively inhibited *Staphylococcus aureus* growth, positioning chitosan as a promising alternative to bone cement for musculoskeletal infections by enabling prolonged drug action without requiring surgical removal (Mahjoub et al. 2024). These findings align with our current study, which observed a greater MRT for the film formulation compared to the conventional formulation.

The *T*_max_ values for the film formulation were significantly greater (*p* < 0.05) than those for the conventional formulation, indicating a slower drug release rate and resulting in prolonged therapeutic effects. These properties support its suitability as a sustained‐release drug delivery system.

Similar to the PLGA/CS‐based intravitreal microimplant for methotrexate (MTX) (Manna et al. 2021), our triple‐layer ENR film demonstrated sustained‐release kinetics with significantly prolonged drug exposure, as evidenced by the increased MRT (196.34 ± 12.82 vs. 6.77 ± 2.45 h in the conventional formulation). Both systems achieved controlled release through a layered design—a hydrophilic core (chitosan for MTX; an analogous polymer in the ENR film) paired with a hydrophobic outer layer (PLGA for MTX; triple‐layer matrix for ENR)—resulting in lower *C*_max_ (0.017 ± 0.003 µg/mL for ENR; 28.88 µM for MTX vs. 400 µM in injections) and extended therapeutic duration (8 weeks for MTX; 144 h for ENR). Notably, both formulations maintained therapeutic concentrations without toxicity, underscoring the efficacy of polymer‐based systems in mitigating burst release and reducing administration frequency. These parallels highlight the broader applicability of layered biodegradable implants for both hydrophilic and lipophilic drugs (Manna et al. 2021).

The cross‐linking process typically enhances the rigidity of the chitosan matrix and hinders the penetration of the release medium into the implant. The resulting cross‐linked film sustains drug release for up to 7 days. Iqbal et al. (2012) similarly reported that non‐glutaraldehyde‐exposed chitosan implants containing tramadol sustained release for 8 days, whereas those exposed to glutaraldehyde for varying intervals sustained release for up to 17 days.

Visual inspections of the implantation sites were conducted frequently post‐implantation, revealing no signs of irritation, infection or inflammatory responses associated with either the administration procedure or the implant itself during the 1‐week study period.

The triple‐layer films prepared in this study comprised two distinct drug delivery systems (Rajgor et al. 2011). The second layer resembled a monolithic structure, whereas the first and third layers functioned as polymeric membranes encasing the drug core in a reservoir‐type system. Specifically, the second layer was applied centrally to the first layer, with both the upper and lower layers remaining drug‐free. Drug diffusion initially occurred from the quarter‐circular release area through both radii during the initial release phase, followed by gradual release through the polymer matrix via diffusion and erosion mechanisms.

Other researchers have also explored various established sustained‐release formulations of ENR. Yu et al. developed a novel thermoreversible in situ‐forming gel using poloxamers for prolonged ENR release. In vivo PK evaluations following a single 20 mg/kg IM injection in pigs demonstrated terminal elimination half‐life (*t*_1/2_ *λz*) and MRT values of 42.92 ± 9.80 and 49.39 ± 8.97 h, respectively. The in vivo performance of the ENR thermoreversible in situ‐forming gel was significantly superior to that of a conventional ENR injection (Yu et al. 2016).

A comparative PK study of injectable ENR in dogs evaluating an in situ‐forming gel versus a conventional injection reported the following parameters: MRT of 45.59 ± 14.05 versus 11.40 ± 1.64 h; area under the concentration–time curve (AUC) of 28.66 ± 15.41 versus 11.06 ± 3.90 µg h/mL; maximum plasma concentration (*C*_max_) of 1.59 ± 0.35 versus 1.46 ± 0.07 µg/mL; time to *C*_max_ (*T*_max_) of 1.25 ± 1.37 versus 1.40 ± 0.55 h; and terminal elimination half‐life (*t*_1/2_ *λz*) of 40.27 ± 17.79 versus 10.32 ± 0.97 h, respectively. These results indicate that the in situ‐forming gel system may extend the ENR dosing interval, thereby reducing administration frequency in long‐term treatments (Li et al. 2015).

The findings of the present study align with those reported in the aforementioned research.

Although multilayer film systems are not novel, recent advances have been made in their preparation and evaluation. The primary advantage of such systems lies in their capacity for targeted local drug delivery at a sustained rate, particularly for postoperative applications intended to reduce infection risks. Postoperative SSIs are an inherent risk associated with any surgical intervention (Hayes et al. 2013). Antibiotic administration represents the most effective strategy for preventing such infections (Periti et al. 1998). Localized antibiotic application offers multiple therapeutic benefits for both treatment and prophylaxis, ensuring high local concentrations at surgical sites that enhance penetration into biofilms and necrotic tissue, ultimately improving bacterial eradication. Furthermore, patient compliance is optimized when antibiotic agents are directly implanted at the surgical site (Hayes et al. 2013).

The film formulation demonstrated gradual release over 144 h; however, ENR plasma levels declined rapidly after 24 h. Several parameters must be considered when designing novel biodegradable systems. The polymer degradation rate in vivo can be influenced by various factors, including fluctuations in body pH or temperature. Additionally, the surface area of the delivery system plays a crucial role in its degradation, as it diminishes during the erosion process. Consequently, changes in the shape of the drug delivery system should be considered during formulation design; it is essential to implement geometrical configurations that maintain a consistent surface area throughout erosion to achieve a more uniform and controlled release profile. These factors may explain the rapid drug release observed within the initial 24 h, followed by a slower release phase over the subsequent 144‐h period.

Antimicrobial agents should be applied locally, informed by knowledge of the structural and biochemical properties of target microorganisms, alongside the pharmacodynamics and PK of the agents, to maximize efficacy and prevent the development of resistance among pathogenic organisms (Aboubakr 2013). The PK of fluoroquinolone antimicrobials have been extensively characterized across various animal species and administration routes (Haritova and Lashev 2009). Establishing PK profiles in animal models is paramount and forms a prerequisite for pharmacological assessments of novel formulations. Successful drug delivery in an animal model represents a significant step toward eventual applications in target animal populations (Wang et al. 2005).

Antimicrobial drugs primarily exert their action through concentration‐dependent mechanisms, demonstrating significant post‐antibiotic effects. They effectively inhibit bacterial growth for hours after plasma concentrations decline below the MIC levels (Otero et al. 2009). McKellar et al. administered danofloxacin and ENR to cattle via recommended dosages and routes used in clinical practice. Both antimicrobials achieved concentrations in exudate, plasma and bronchial secretions exceeding the MIC_90_ values for common bovine pathogens such as *Pasteurella multocida*, for which the reported MIC_90_ for both agents is 0.06 µg/mL (McKellar et al. 1999). The concentration of ENR measured for the film formulation was 0.212 µg/mL at 144 h post‐administration. Furthermore, the PK behaviour of the ENR film following SC administration at 33.3 mg/kg body weight in rabbits demonstrated that plasma ENR concentrations remained above the MIC level reported for susceptible bacteria (0.06 µg/mL) throughout the 144‐h monitoring period (McKellar et al. 1999).

## 5 Conclusion

In summary, comparative PK analyses indicate that the ENR film formulation effectively reduces drug elimination and extends the residence time of ENR in systemic circulation. The primary advantage of this system lies in its capacity for targeted local drug delivery at a sustained rate, making it particularly suitable for postoperative applications aimed at infection prevention. Overall, the ENR film demonstrates considerable promise as a sustained‐release preparation, warranting further investigation for potential applications in treating susceptible microbial infections in veterinary practice.

## Author Contributions

Sakineh Khanamani Falahatipour, principal investigator for the project, responsible for the overall conceptualization, design and execution of the research, managed the project timeline, conceived of the study, designed the experiment, collected and analysed the data, wrote the initial draft of the manuscript and approved the final version. Ali Rassouli assisted with data collection, provided crucial theoretical insights into the methodology, performed statistical analysis and data curation, validation, supervision, contributed to the interpretation of the results and reviewed and edited the manuscript. Hamid Akbari Javar provided critical feedback on the study design, provided crucial theoretical insights into the methodology, supervised the data collection process, data curation, validation, formal analysis, visualization and helped draft the manuscript. Yalda Hosseinzadeh Ardakani, responsible for the overall conceptualization, provided crucial theoretical insights into the methodology, contributed significantly to the interpretation of the findings and data curation, validation, formal analysis, supervision, visualization, reviewed and revised the manuscript multiple times and assisted with the preparation of the figures and tables.

## Disclosure

We, the undersigned, confirm that all authors meet the journal's requirements for authorship and have made substantial contributions to the conception, design, execution or interpretation of the work described in this manuscript. All authors have read and approved the final manuscript and agree to be accountable for all aspects of the work. We confirm that the manuscript is original, has not been published elsewhere and is not currently under consideration by any other journal.

## Ethics Statement

The authors declare that this study was approved by and conducted in accordance with the ethical requirements of the University of Tehran. The study protocol received approval from the Ethics Committee of the Faculty of Veterinary Medicine, University of Tehran (Approval No: 7506006‐6‐10). The authors confirm that all procedures adhered to internationally recognized standards for the protection of animals used in scientific research.

## Conflicts of Interest

The authors declare no conflicts of interest.

## Peer Review

The peer review history for this article is available at https://publons.com/publon/10.1002/vms3.70618.

## Data Availability

The datasets generated and/or analysed during the current study are available at https://scholar.google.com/citations?user=8qz6Hi8AAAAJ&hl=en.
